# Factors Predisposing to Maxillary Anchorage Loss: A Retrospective Study of 1403 Cases

**DOI:** 10.1371/journal.pone.0109561

**Published:** 2014-10-09

**Authors:** Hong Su, Bing Han, Sa Li, Bin Na, Wen Ma, Tian-Min Xu

**Affiliations:** 1 Department of Orthodontics, Peking University School and Hospital of Stomatology, Beijing, China; 2 Department of Stomatology, Shenzhen People's Hospital, Shenzhen, China; 3 Peking University School and Hospital of Stomatology, Beijing, China; University of Palermo, Italy

## Abstract

Anchorage loss is very disturbing for orthodontists and patients during orthodontic treatment, which usually results in bad treatment effects. Despite the same treatment strategy, different patients show different tendencies toward anchorage loss, which influences the treatment results and should preferably be predicted before the treatment is begun. However, relatively little research has been conducted on which patients are more likely to lose anchorage. The mesial tipping of the first molar marks the onset of anchorage loss, and changes in the angulation of the first molar are closely related to anchorage loss. This cross-sectional study aimed to determine how the mesiodistal angulation of the upper first molars changes during general orthodontic treatment and to identify the individual physiologic factors leading to these changes in a large sample of 1403 patients with malocclusion. The data indicate that the upper first molars tend to be tipped mesially during orthodontic treatment, and this constitutes a type of anchorage loss that orthodontists should consider carefully. Compared to treatment-related factors, patients' physiologic characteristics have a greater influence on changes in the angulation of the upper first molars during orthodontic treatment. The more distally tipped the upper first molars are before treatment, the more they will tip mesially during treatment. Mesial tipping of the upper first molars, and therefore, anchorage loss, is more likely to occur in adolescents, males, patients with class II malocclusion and patients who have undergone maxillary premolar extraction. This finding is of clinical significance to orthodontists who wish to prevent iatrogenic anchorage loss by tipping originally distally tipped upper molars forward, and provides a new perspective on anchorage during orthodontic treatment planning.

## Introduction

As early as 1728, the French dental pioneer Fauchard proposed that if mechanical forces were used during dental treatment, adequate tooth anchorage must be provided [1]. For contemporary orthodontists, anchorage is crucial, and the method of controlling anchorage is one of the major concerns during the development of a treatment plan. To this end, orthodontists have designed a variety of intraoral or extraoral devices, such as Nance palatal arch, lingual arch, transpalatal arch, headgear and temporary anchorage devices to strengthen anchorage [1].

Anchorage is often required to retract protruding anterior teeth in the upper jaw or to relieve maxillary crowding. Unfortunately, owing to certain anatomical characteristics, anchorage loss occurs much more easily in the upper jaw than in the lower jaw. This dilemma compels us to consider the role of the upper first molars (UMs) in anchorage control. Not only the displacement, but also the angulation of the UMs affects anchorage. Since Andrews proposed his famous “Six Keys to Normal Occlusion” (the second of which was angulation) in 1972 [2], angulation changes have been examined in various studies. Roth [3], [4] argued that maintaining proper mesiodistal inclinations of teeth is necessary, for not only dental alignment but also the long-term stability of orthodontic treatments. Orthodontists once tried to achieve “proper” upper molar angulation; today, they seem to accept the fixed angulation achieved using pre-adjusted buccal tubes or brackets. This standard prescription is applied to a wide variety of malocclusions, even though the different initial molar angulations and their changes during orthodontic treatment presumably have a significant effect on anchorage and on the results of treatment.

Mesial tipping of the UMs is a common observation during orthodontic treatment [5], [6], [7], [8], [9], [10], [11]. For patients requiring maximal anchorage, mesial tipping of the UMs means anchorage/space loss, which often leads to occlusal plane changes and bad treatment results. In contrast, distal tipping of the UMs seems to be beneficial. Accordingly, several orthodontic techniques (e.g., Tweed edgewise, Begg light-wire) use molar tip-back bends on stainless steel archwires to tip the first molars backward in order to reinforce anchorage and prevent mesial UM tipping. Most studies have focused on molar linear displacement during the treatment of anterioposterior discrepancy [7], [11], [12], [13], [14]; few have examined molar tipping changes in general orthodontic treatments.

It is particularly disturbing that while orthodontists try to control anchorage using sophisticated methods and appliances, different patients show different responses to a given treatment. In fact, in some patients, the UMs are nearly immobile throughout treatment, whereas in others, the UMs begin to tip forward rapidly at the start of treatment. Although orthodontists would like to determine in advance which patients are more likely to lose anchorage, the problem has not yet been solved. Most studies have focused on the type of appliance or method of anchorage reinforcement [15], [16], [17], [18], [19], [20], [21], [22], [23], [24]; few have examined patients' natural physiologic characteristics that might affect molar anchorage. Thus, the factors that cause continuous anchorage loss are not completely clear.

The aim of this retrospective cross-sectional study is to investigate how the mesiodistal angulation of the UMs changes during general orthodontic treatments and to identify the factors related to these changes, especially, the physiologic characteristics of the patients.

## Materials and Methods

### Sample sources

The study sample was selected from a database comprising >11,000 patients who had finished orthodontic treatment between 1997 and 2005 at Peking University School and Hospital of Stomatology (PKUSS; Beijing, China). All the records/information of the patients were anonymized and de-identified prior to analysis. The ethics committee of PKUSS approved the study protocol (protocol number: PKUSSIRB-2013020; approval date: February 6, 2013).

#### Inclusion criteria

Han nationality; no hereditary diseases; complete medical records; fixed-appliance treatment; and lateral cephalograms taken before and after treatment, all with the same X-ray machine.

#### Exclusion criteria

Unfinished treatment or a need for re-treatment; treatment without fixed appliances; missing maxillary first molars or a treatment plan calling for the extraction of the maxillary first molars; and orthognathic surgery.

The study cohort consisted of a total of 1403 patients who met the selection criteria. Considering the study objectives, we analyzed patients who had different physiologic characteristics or had undergone different treatments. The composition of the study subjects has been described in the Results section.

### Measurements

Five senior orthodontic residents who were blinded to the goals of our study recorded the medical information and made the cephalometric measurements. Variables chosen from the medical records and the cephalometric parameters used in this study are listed below.

### Medical records

Physiologic characteristics: age, gender, molar relationship, deep overjet, deep overbite, openbite and upper crowding.

Treatment options (presence or absence): premolar extraction, second molar treatment, flat bite plate, occlusal plate, pendulum, transpalatal arch (TPA); Nance arch, maxillary protraction, headgear and palatal expansion.

**Figure 1:**
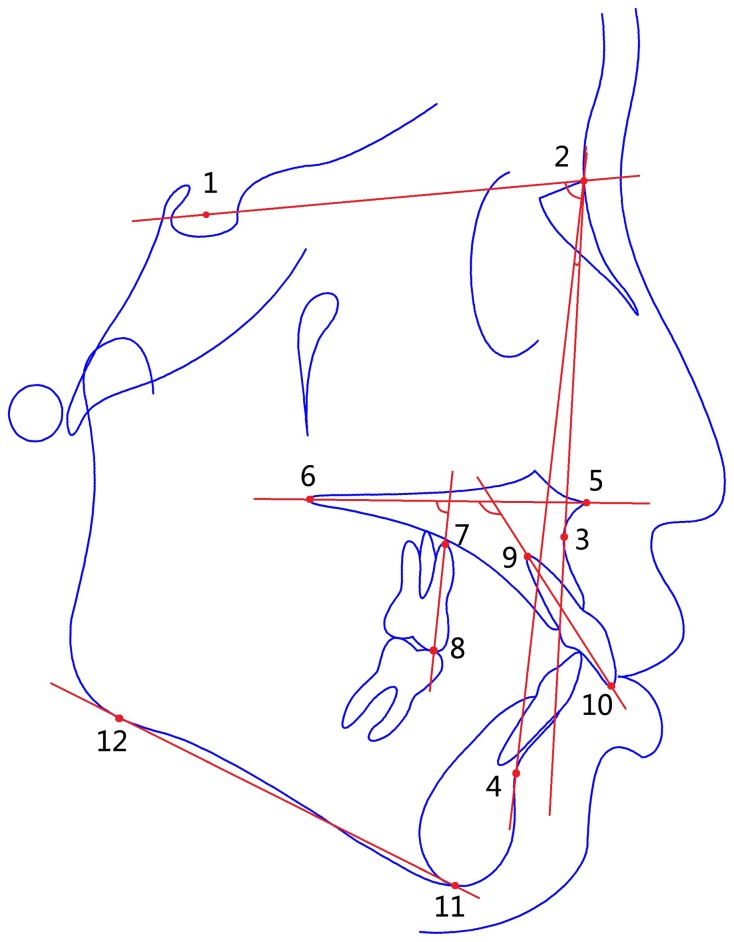
Cephalometric landmarks. 1. Sella (S); 2. Nasion (N); 3. Subspinale (A); 4. Supramentale (B); 5: Anterior nasal spine (ANS); 6: Posterior nasal spine (PNS); 7: Apex of the mesiobuccal root of UM (UMA); 8: Mesiobuccal cuspid of UM (UMC); 9: Apex of the root of upper incisor (UIA); 10: Edge of upper incisor (UIE); 11: Menton (Me); 12: Point of tangency of mandibular plane.

Lateral cephalograms were provided by the Department of Radiology of PKUSS. To control for magnification, all headfilms were taken with the same cephalostat and the same object–film distance. After the cephalograms were scanned, landmarks were located three times each by three senior residents. Bilateral images were split. According to Baumrind's study of the reasonable range of landmark positions [25], [26], [27], outliers (which, if present, are mostly caused by inadvertent clicking on the screen) can be automatically detected by a customized software and were checked by the same judge. The average of the nine measurements of landmark positions was used in subsequent calculations by the customized software. The palatal plane (PP) and Downs' mandibular plane (MP) [28] were located. A line traced from the tip of the mesiobuccal cusp to the apex of the mesiobuccal root of UM served as a measure of the long axis. The angulation of this axis in relation to the PP is abbreviated to UM/PP. Change in UM/PP during orthodontic treatment is the dependent variable of our study. Cephalometric landmarks are illustrated in Figure 1. The various measures and their interpretation are summarized in Table 1. The following suffixes will be employed to modify the abbreviations: before treatment, “-1”; after treatment, “-2”; and treatment change, “-12”.

**Table 1:** Cephalometric variables and definitions.

Variable	Definition
	Angulation
UM/PP	Inferoposterior angle of the axis of upper first molar with the palatal plane
SNA	Inferoposterior angle of NA with SN
SNB	Inferoposterior angle of NB with SN
ANB	SNA angle minus SNB angle
MP/SN	Anteroinferior angle of the mandibular plane with SN
UI/PP	Inferoposterior crossing angle of upper incisor axis with the palatal plane
	Linear
UIE-PP	Perpendicular distance from UIE to the palatal plane
UMC-PP	Perpendicular distance from UMC to the palatal plane
UIE-PP/UMC-PP	Ratio of UIE-PP to UMC-PP

### Statistical analyses

Data were analyzed using SPSS v16.0 (SPSS, Chicago, IL, USA). The type I error rate was set at *P* <0.05. For the classificatory variables, independent-samples *t*-tests and analysis of variance (ANOVA) were used to analyse between-categories differences. Significant classificatory variables and common cephalometric variables were used for multiple linear regression analysis (stepwise method). UM/PP-12—symbolizing upper first molar anchorage loss—was the dependent variable.

## Results

### UM/PP-12 and physiologic characteristics

**Figure 2:**
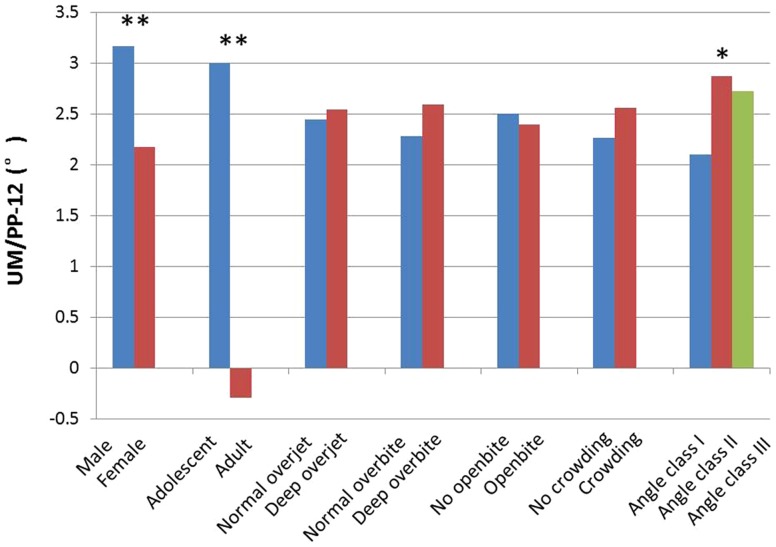
Analysis of UM/PP-12 in groups with different physiologic factors. The change in UM angulation during orthodontic treatment (i.e., the dependent variable) significantly differed with gender, age and molar relationship.

On average, the maxillary first molars tipped forward 2.50° during treatment. The results of independent-samples *t*-tests and ANOVA are summarized in Table 2 and Figure 2. There were statistically significant differences for gender (male, 3.17°; female, 2.18°), age (adult, -0.29°; adolescent, 3.00°) and molar relationship (Class II, 2.87°; Class I, 2.10°). The average change in UM/PP among adults was close to 0°, which means that the UM/PP in adults was nearly unchanged throughout orthodontic treatment. In addition, the presence or absence of deep overjet, deep overbite, openbite and upper crowding did not significantly affect the UM/PP.

**Table 2:** Upper anchorage loss as a function of selected physiologic characteristics.

Factor		Number	UM/PP-12		
			Mean	Standard Deviation	*P* value
Gender	Male	457	3.17	5.31	<0.01**
	Female	946	2.18	5.11	
Age	Adolescent	1190	3.00	5.11	<0.01**
	Adult	213	−0.29	4.79	
Deep overjet	No	637	2.45	5.35	0.72
	Yes	766	2.55	5.07	
Deep overbite	No	410	2.28	4.91	0.31
	Yes	993	2.59	5.31	
Openbite	No	1375	2.50	5.20	0.92
	Yes	28	2.40	5.40	
Upper crowding	No	287	2.27	5.27	0.39
	Yes	1116	2.56	5.18	
Molar relationship	Class I	635	2.10	4.98	0.03*
	Class II	547	2.87	5.26	
	Class III	221	2.72	5.56	

*: *P*<0.05;

**: *P*<0.01.

### UM/PP-12 and treatment-related factors

**Figure 3:**
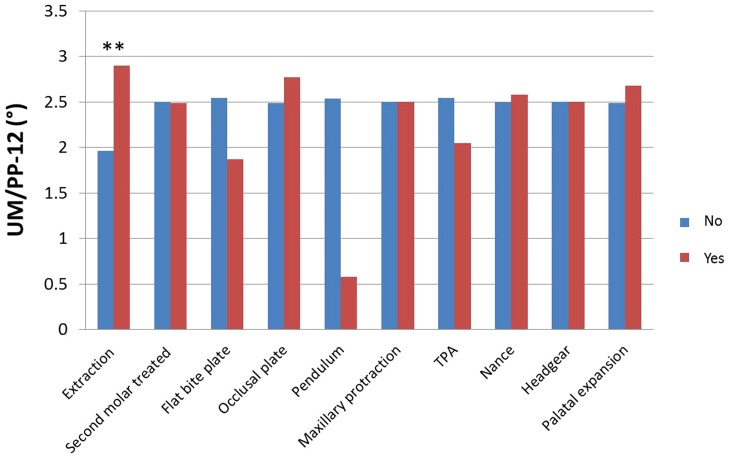
Analysis of UM/PP-12 in groups with different treatment strategies. The change in UM angulation during orthodontic treatment (i.e., the dependent variable) significantly differed between patients with and without maxillary premolar extraction.

The results of independent-samples *t*-tests for the analysis of UM/PP-12 and treatment-related factors are shown in Table 3 and Figure 3. There were very significant differences in UM/PP-12 for premolar extraction (extraction 2.90° *vs.* non-extraction 1.96°). The UMs tipped more mesially in patients who had undergone maxillary premolar extraction than in those who had not. The presence or absence of second molar treatment, flat bite plate, occlusal plate, pendulum, TPA, Nance arch, maxillary protraction, headgear and palatal expansion did not significantly affect the UM/PP.

**Table 3:** Upper anchorage loss as a function of selected treatment-related factors.

Treatment Variable	Alternative	Number	UM/PP-12		
			Mean	Standard Deviation	*P*
Premolar extraction	No	601	1.96	5.18	<0.01**
	Yes	802	2.90	5.17	
Second molar treatment	No	1170	2.50	5.19	0.96
	Yes	233	2.48	5.24	
Flat bite plate	No	1306	2.55	5.19	0.22
	Yes	97	1.87	5.31	
Occlusal plate	No	1342	2.49	5.22	0.68
	Yes	61	2.77	4.68	
Pendulum	No	1377	2.54	5.20	0.06
	Yes	26	0.58	4.82	
Maxillary protraction	No	1371	2.50	5.13	0.99
	Yes	32	2.50	7.62	
TPA	No	1287	2.54	5.24	0.32
	Yes	116	2.05	4.65	
Nance arch	No	1320	2.50	5.16	0.89
	Yes	83	2.58	5.70	
Headgear	No	1062	2.50	5.09	0.99
	Yes	341	2.50	5.51	
Palatal expansion	No	1305	2.49	5.16	0.72
	Yes	98	2.68	5.66	

**: *P*<0.01.

### UM/PP-12 and multivariate linear regression analysis

We tested the influence of frequently used cephalometric variables on UM/PP-12. The descriptive statistics are shown in Table 4. To compare the impacts of various factors on the UM/PP-12, we selected the statistically significant factors mentioned in Tables 2 and 3, and the frequently used cephalometric variables listed in Table 4. These factors were used to perform a multivariate linear regression analysis (Table 5). Seven variables were brought into the regression equation (R^2^ = 33.8%). Among them, the angulation of the UMs before treatment (standardized coefficient: -0.655) was the most influential factor with respect to the change in UM/PP during treatment.

**Table 4:** Frequently used cephalometric variables.

Measure	Mean	Standard deviation
ANB-1 (°)	4.18	2.79
SNA-1 (°)	82.10	3.43
SNB-1 (°)	77.95	3.79
MP/SN-1 (°)	37.42	5.79
UI/PP-1 (°)	119.00	8.02
UIE-PP-1 (mm)	31.52	2.94
UM/PP-1 (°)	79.62	5.87
UMC-PP-1 (mm)	24.65	2.80
UIE-PP/UMC-PP-1 (ratio)	1.29	0.11

*: *P*<0.05;

**: *P*<0.01.

**Table 5:** Results of multiple linear regression analysis of UM/PP-12 and correlated variables.

	Unstandardized Coefficients	Std. Error	Standardized Coefficients	t	P
(Constant)	55.274	4.295		12.868	<0.001
UM/PP-1	−0.580	0.024	−0.655	−24.008	<0.001
UIE-PP/UMC-PP-1	−7.770	1.325	−0.162	−5.864	<0.001
ANB-1	−0.379	0.049	−0.204	−7.818	<0.001
Premolar extraction	1.623	0.248	0.154	6.558	<0.001
UIE-PP-1	−0.154	0.041	−0.087	−3.732	<0.001
Gender	−0.957	0.248	−0.086	−3.852	<0.001
SNA-1	0.116	0.036	0.077	3.207	0.001

## Discussion

During orthodontic treatment, the mesial tipping of the UM marks the onset of anchorage loss; however, this tipping is rarely considered in anchorage planning. To date, research in this area is scarce and usually limited by sample size, selection bias, study design, etc. The present cross-sectional study was carefully designed to avoid these shortcomings by employing a sample of more than 1,400 cases and a comprehensive analysis of many aspects of these treatments. The results are surprising, in that people with certain initial characteristics are more likely to lose anchorage by way of UM angulation changes.

### UM/PP-12 and individual physiologic factors

#### Growth

The study showed that mesial tipping of the UMs during orthodontic treatment was a common phenomenon, regardless of the presence or absence of a history of maxillary premolar extraction, and occurred even in the presence of anchorage auxiliaries. Orthodontic treatment is invariably long, typically lasting for more than 2 years. During this period, growth itself may bring about changes in the angulation of the UMs. In the study by Iseri and Solow [29], continued downward–forward eruption of the UMs was observed even until 25 years of age, though the velocity was very slow by then. If this is the case, growth may be a double-edged sword for upper anchorage preservation. The natural tendency of forward tipping of the UMs would cause a physiologic anchorage loss, especially, when met with some accelerating mechanical force during treatment.

#### Age

Most orthodontic patients are adolescents. Table 2 and Figure 2 show that on average, the UMs of adolescents tipped 3.0° mesially, whereas those of adults basically stayed still, with a statistically significant difference between these two patient groups. This finding is in accordance with the results of our former prospective randomized clinical trial [8] and McKinney and Harris [30], both of which showed a greater tendency to anchorage loss in adolescents. Probably a dentoalveolar compensation of the UMs for the excess mandibular growth commonly occurs in growing patients [31].

Adolescence is the second peak in growth and development. The mesial tipping movement of the UMs is remarkable in this stage. Many longitudinal studies [31], [32], [33], [34], [35] have shown that the UMs gradually tilt mesially in teenagers. Iseri and Solow [29] thought the eruption of UMs at about 12 years changed in a forward direction due to the resulting compensatory dentoalveolar adaptation to the continued forward growth of the mandible, and the peak velocity occurred at 12 years of age among females. Tsourakis and Johnston [31] also found a connection between mandibular excess and mesial UM movement in growing patients. Therefore, in adolescent patients, molar anchorage loss during treatment might be attributable to two factors: orthodontic force (mechanical anchorage loss) and growth-related changes and/or molar drifting after extraction (physiologic anchorage loss). The latter is usually overlooked by orthodontists.

**Figure 4:**
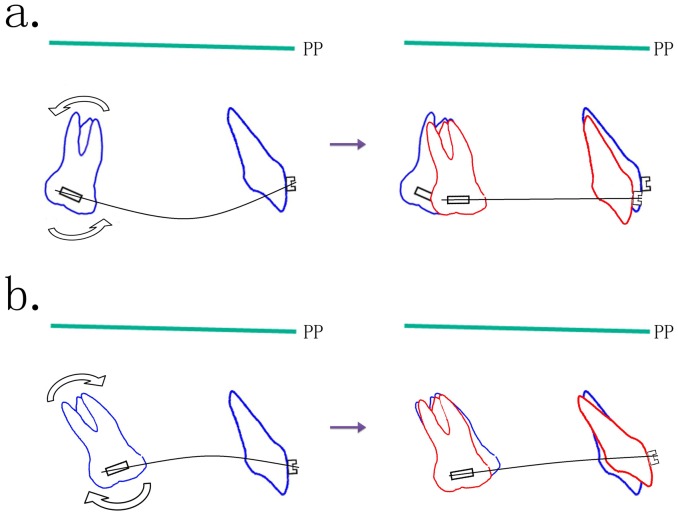
Simulations of the early treatment of patients with different initial upper molar (UM) angulations. Patients with different UM angulations would show different responses to the same treatment. a. In patients with more distally tipped UMs, the mesial tipping moment from the deflected NiTi archwire into the 0° buccal tube would tip the molars forward to occupy the extraction space, and thus, constitute anchorage loss. b. In patients with extremely mesially tipped UMs, the moment from a straight archwire in 0° buccal tubes would tip the UMs backward.

Our previous cross-sectional study [36] has found that the UMs tend to be distally tipped in younger patients compared to their position in older patients. However, most orthodontists treat patients with straight-wire appliances in clinics. When a straight NiTi wire is inserted into a 0° buccal tube on molars with different angulations, it may tip a backward tipped molar forward and a forward tipped molar backward (Figure 4). The forward tipping moments would result in accelerated anchorage loss, even in the alignment stage. This phenomenon may explain why anchorage loss is more likely to occur in adolescents, and the natural backward tipping of juvenile UMs might be the leading cause of anchorage loss in adolescent patients.

#### Gender

Table 2 and Figure 2 show that remarkably, gender has an impact on the UM/PP-12. During orthodontic treatment, the mean mesial tipping of UMs for boys in our study was significantly greater than the mean mesial tipping for girls. This result is in agreement with that of our prospective randomized clinical trial [8], which showed that male patients are more prone to anchorage loss than are female patients. Similarly, greater mesial tipping of the maxillary molars in boys was found in the study by McKinney and Harris [30]. This could be attributable to the later growth spurt in males. Compared to females, males may show a greater degree of backward tipping of the UMs at the same chronological age, and consequently, the UMs may be more likely to tip forward under the same treatments. The reason could also be that boys grow more than girls; hence, more mesial dentoalveolar compensation of the UMs may occur when the mandible grows.

#### Malocclusion type

From Table 2 and Figure 2, it may be seen that there are statistically significant differences between patients with different molar relationships: UMs will be tipped more mesially during treatment in class II patients. That is to say, class II patients are more likely to lose anchorage. Björk and Skieller [35] stated that tooth position changes constantly to compensate for the change in jaw position during growth (dentoalveolar compensation). Our previous cross-sectional study [36] found that patients with class II malocclusion had the most distally tipped UMs. Similar results were reported by Kim et al. [34] and Martinelli et al. [32]. Therefore, as shown in Figure 4a, the initial backward compensations of the UMs are easily overcome by straight archwires in unified 0° buccal tubes, leading to a worsened distal molar relationship, early anchorage loss and possibly a reduction in space for anterior retraction. Class II malocclusions, however, usually symbolized by convex profiles, deep overjet and overbite, and hence, have a stronger requirement for anchorage. This contradiction would make orthodontists turn to headgears and miniscrews for maximum anchorage control or even molar distalization in such patients. This lose-anchorage-then-reinforce-it process is an unnecessary to and fro movement, which not only extends treatment time but also increases patient discomfort. This phenomenon of molar crown tipping in a straightwire system using preangulated brackets has also been reported by McKinney and Harris [30]. It would seem to be an unnecessary iatrogenic anchorage loss at the start of treatment.

### UM/PP-12 and treatment-related factors

From Table 3 and Figure 3, we can conclude that the only statistically significant treatment-related factor in terms of anchorage loss is upper premolar extraction, a strategy that leads to more mesially tipped molars. This result is in agreement with those of other studies [5], [6], [10], which have showed that molar mesial tipping is more likely to occur in patients who have undergone premolar extraction. It is worth noting that UM mesial tipping also occurs in non-extraction patients (UM/PP-12≈2.0°), a finding which implies that mesial tipping is not induced only by “tiebacks” or space closure. Even for non-extraction treatments, UM mesial tipping is influenced by factors such as the individual's growth stage and original molar angulation.

### UM/PP-12 and multiple regression analysis

Regression analysis (Table 5) showed that the most influential factor for UM/PP-12 is the initial angulation of the UMs before treatment. Its influence is overwhelming and is determined by the morphological characteristics of the dentition, rather than the use of elastic force for space closure, as is conventionally believed. The negative standardized coefficient indicates that the more the UMs are naturally backward tipped before treatment, the more they will be tipped mesially, or in other words, the more anchorage loss will happen, during orthodontic treatment. It is known that UMs with different pretreatment angulations compensate differently for different malocclusions or at different growth stages [33], [34], [36], [37], [38], [39], [40]; for the sake of anchorage control, the initial UM angulation should be evaluated and taken advantage of when developing treatment plans.

To sum up, our results challenge the traditional belief that molar anchorage loss is caused only by the elastic forces used for space closure, and provide a new perspective on anchorage control. We found that the compensation of UM angulation for mandibular growth, one of the physiologic characteristics of natural dentition, plays a more important role in anchorage control. It is reasonable to conclude that since modern straight wire appliances use the same 0° buccal tube to treat molars with different angulations, they elicit different responses in different patients. Anchorage loss is more likely to happen in certain groups such as adolescents, patients with convex class II malocclusion and/or high angles whose UMs tend to tip backward before treatment [36]. To overcome iatrogenic anchorage loss in these patients and to prevent physiologic anchorage loss during treatment, we recommend the Cross Buccal Tube (XBT) [36], [41], which consists of a −25° round tip-back tube and a −7° rectangular tube designed for providing 24-h tip-back moments during the whole treatment period, both in the round thin arch wire stage and the thick rectangular arch wire stage.

### Conclusions

The maxillary first molars tend to be tipped mesially during orthodontic treatment, and this constitutes a type of anchorage loss of which orthodontists should be aware. Compared to treatment-related factors, the patients' physiologic characteristics have a greater influence on changes in the angulation of the UMs during orthodontic treatment. The initial UM angulation is the most significant factor contributing to anchorage loss. The more distally tipped the UMs are before treatment, the more they will tip mesially during treatment.

Mesial tipping of the UM, and therefore, anchorage loss, is more likely to occur in certain groups, such as adolescents, males, those with class II malocclusion and/or those who have undergone maxillary premolar extraction. Orthodontists should take measures to avoid possible iatrogenic anchorage loss and develop more customized treatment plans for such patients.

## Supporting Information

Data S1 **The original data of the study, including the physiologic factors and the treatment strategies of all the patients.**
(XLSX)
